# Human Papillomavirus and Prostate Cancer: A Controversial Association

**DOI:** 10.3390/pathogens15101070

**Published:** 2026-10-09

**Authors:** Sara Sá, Mariana Sousa, Ana Cláudia Pereira, Pilar Baylina, Fátima Cerqueira

**Affiliations:** 1 FP-BHS—Biomedical and Health Sciences Research Unit, FP-I3ID—Institute for Research, Innovation, and Development, FFP—Fundação Fernando Pessoa, 4249-004 Porto, Portugalanacpereira@ufp.edu.pt (A.C.P.); pilarbaylina@ess.ipp.pt (P.B.); 2 Epidemiology, Outcomes, Economics and Management in Oncology Group, IPO Porto Research Center (CI-IPO Porto), Portuguese Oncology Institute of Porto ( IPO Porto), 4200-072 Porto, Portugal; mariana.p.sousa@ipoporto.min-saude.pt; 3 Experimental Pathology and Therapeutics Group, Research Center (CI-IPOP), Portuguese Oncology Institute ( IPO Porto), 4200-072 Porto, Portugal; 4 RISE—Associate Laboratory (Health Research Network), 4200-319 Porto, Portugal; 5 Porto Comprehensive Cancer Centre Raquel Seruca (Porto.CCC), 4200-072 Porto, Portugal; 6 FCS—Faculty of Health Sciences, Fernando Pessoa University, 4249-004 Porto, Portugal; 7 School of Health, Technical University of Porto, 4200-072 Porto, Portugal; 8 RISE-Health, University Fernando Pessoa, 4200-150, Porto, Portugal; 9 Molecular Oncology and Viral Pathology Group, Research Center (CI-IPOP), 4200-072 Porto, Portugal

**Keywords:** human papillomavirus, prostate cancer, viral infection, oncogenic virus

## Abstract

Human papillomavirus (HPV) is a sexually transmitted virus which infection can be asymptomatic or appear as mucosal lesions. HPV infection is estimated to contribute to approximately 5% of all cancers worldwide and is strongly associated with the development of most cervical cancers, as well as a substantial proportion of oropharyngeal, penile, and vulvar cancers. The viral HPV proteins E6 and E7 promote the development of tumour cells by disturbing the cellular signalling pathways involving the retinoblastoma protein (pRB) and cellular tumour antigen p53. Due to the established role of HPV in several anogenital cancers, a possible association between HPV infection and prostate cancer (PCa) risk has also been investigated. Recent studies and meta-analyses have reported an association between HPV infection and PCa in some populations. However, the available evidence remains inconsistent, and HPV infection cannot currently be considered an established risk factor for PCa. Further studies are needed to determine whether HPV infection plays a causal role in prostate carcinogenesis and whether HPV detection has clinical or prognostic utility in PCa.

## 1. Introduction

Human papillomavirus (HPV) is a virus that can infect the skin and the mucous membranes of the human body. With 200 types known to exist, HPV is one of the most prevalent sexually transmitted diseases (STDs) in the world [1,2], with asymptomatic characteristics or clinical manifestations as genital warts and other mucosal lesions. HPV infection is a serious public health problem, not only due to its dermatological manifestations but also because persistent infection with high-risk HPV types can contribute to carcinogenesis through the disruption of cellular pathways involved in cell-cycle regulation, apoptosis, and genomic stability tumour [3]. In fact, over the last years, HPV has become well established as an etiological agent of several anogenital and oropharyngeal cancers, including cervical, anal, penile, vulvar, vaginal, and a proportion of oropharyngeal cancers. This established association has raised interest in whether HPV infection may also be involved in the development of prostate cancer (PCa) [1,2,3].

This review presents a summary of the existing evidence on HPV biology and detection, its well-established role in the development of cancer, and especially on the current evidence regarding the association between HPV infection and PCa. Special attention is paid to the methodological approaches employed for detecting HPV in prostate tissue and in non-invasive biological samples, as well as to the limitations that, at present, prevent any definite conclusions about a causal role for HPV in the development of prostate cancer.

## 2. Review Methodology

### 2.1. Search Strategy and Data Sources

To identify and summarize published evidence regarding the association between HPV infection and PCa, a structured literature search was performed in PubMed. The search was conducted using the Medical Subject Headings (MeSH) terms “Human Papillomavirus Viruses” AND “Prostatic Neoplasms”. The search included studies published between 2005 and July 2026. The final search was performed on 17 August 2026.

### 2.2. Eligibility Criteria and Study Selection

Studies were considered eligible if they investigated the association between HPV infection and PCa, including HPV detection, infection, seropositivity, or its potential association with PCa risk or clinicopathological characteristics. Studies were excluded if they were narrative reviews, experimental in vitro studies, bioinformatics or methodological studies, or did not investigate the association between HPV infection and PCa.

The retrieved records were screened based on their titles and abstracts according to the predefined eligibility criteria. Records considered potentially relevant were assessed for eligibility. A total of 27 records were identified through the PubMed search. Following screening, four records were excluded for not meeting the eligibility criteria: one narrative review, one experimental in vitro study, one bioinformatics/methodological study, and one study that did not investigate the association between HPV infection and PCa. A total of 23 studies were included in the qualitative synthesis. The main characteristics and findings of the included studies are summarized in Table 1 and Table 2.

### 2.3. Data Extraction and Synthesis

Data from the included studies were extracted and organized according to publication year, study aim, study population, methodology, and main outcomes. The extracted information was summarized in Table 1 and Table 2. Due to the heterogeneity of the included studies regarding study populations, biological samples, HPV detection methods, and reported outcomes, the findings were synthesized qualitatively.

## 3. HPV Biology and Oncogenic Mechanisms

### 3.1. HPV Structure and Taxonomy

HPVs are divided into different genera such as alpha, beta, mu and gamma. Within each genus, HPVs are further classified into species and individual types based primarily on nucleotide sequence differences in the L1 open reading frame (ORF) [27,28,29].

HPV is a small, non-enveloped virus, with around 50 to 55 nm of diameter [30], displaying a circular double-stranded DNA genome of approximately 7.9 kb [31]. The viral genome is associated with cellular histones and is enclosed within an icosahedral capsid composed of 72 L1 pentamers (capsomers) [32].

The HPV genome consists of a long control region (LCR) and early and late coding regions. The LCR is a non-coding region that contains both the origin of DNA replication and the elements that regulate transcription [33]. The HPV genome encodes early and late proteins, which are expressed at different stages of the viral life cycle and have distinct functions in viral replication, persistence, and interaction with host cells [30]. The early region encodes the proteins E1, E2, E4, E5, E6, and E7, which are mainly involved in viral genome replication, maintenance, and modulation of host–cell functions. The late region encodes the structural capsid proteins L1 and L2 [31,34].

### 3.2. HPV Early and Late Proteins

Among the early proteins, E6 and E7 have particular relevance to HPV-associated carcinogenesis due to their ability to interfere with key cellular tumour-suppressor pathways [31,34].

E1 is the most conserved HPV protein and the only viral protein with known enzymatic activity. It contains functional domains involved in nuclear localization, recognition of the viral origin of replication, and helicase/ATPase activity. E1 interacts with E2 and recruits cellular replication factors to initiate and maintain viral DNA replication [31,34].

E2 regulates viral transcription and replication through its interaction with viral DNA and E1. Depending on its expression level and binding sites, E2 can activate or repress viral transcription. Importantly, disruption or loss of E2 function may result in increased E6 and E7 expression, which has been associated with HPV-related carcinogenesis [35,36,37].

E4 is produced through alternative splicing and is mainly expressed during the productive stages of the HPV life cycle. It has been associated with disruption of keratin organization and may contribute to viral genome amplification and virion release [38,39,40].

E5 is a hydrophobic transmembrane protein that can modulate several cellular signalling pathways. In particular, HPV16 E5 has been associated with increased epidermal growth factor receptor (EGFR) signalling, modulation of VEGF expression, alteration of MHC class I expression, and inhibition of apoptotic pathways. These effects may contribute to cell proliferation, immune evasion, and persistence of infected cells [30,41,42,43,44,45].

E6 and E7 are the major HPV oncoproteins and play a central role in HPV-associated carcinogenesis. E6 promotes the degradation of the tumour suppressor p53, whereas E7 interferes with the retinoblastoma protein (pRb) pathway, contributing to deregulated cell-cycle progression. The molecular mechanisms underlying the oncogenic activity of E6 and E7 are discussed in greater detail in the following section [46,47,48,49,50].

The late region encodes two structural proteins, L1 and L2, which form the HPV capsid. L1 is the major capsid protein, whereas L2 is the minor capsid protein. HPV types are classified according to differences in the nucleotide sequence of the L1 gene [31].

L1 forms pentameric structures that constitute the external surface of the viral capsid and participates in the initial interaction between the virus and host–cell receptors [31,51]. L2 is involved in viral entry and intracellular trafficking and is relatively conserved among HPV types [52]. L1 can self-assemble into virus-like particles (VLPs), which form the basis of currently available prophylactic HPV vaccines [53].

## 4. HPV Detection Methods

### 4.1. Conventional Detection Methods

HPV detection may be carried out by means of molecular or histopathological methods, with the choice depending on the type of biological specimen and the aim of the analysis. Nucleic acid amplification tests, especially polymerase chain reaction (PCR), are commonly employed for the detection and typing of HPV DNA. With tissue specimens, both PCR and in situ hybridization (ISH) can be used to detect viral DNA, while the detection of HPV RNA, specifically that of the E6/E7 transcripts, may offer further evidence of an actively transcribing infection. Immunohistochemical detection of p16^INK4a^ may also be used as a supplementary marker of HPV-associated oncogenic activity, although its interpretation is dependent on the context [6,54]. In the case of prostate cancer, prostate tissue has been the primary biological sample studied for the detection of HPV. Nevertheless, tissue-based methods are invasive and can be restricted by the availability and quality of the tissue samples. Moreover, the mere detection of HPV DNA does not prove that there is an actively transcribing infection or that HPV causes prostate carcinogenesis. For these reasons, alternative methods that are able to detect viral nucleic acids in samples obtained by minimally invasive means might be of interest [5,6].

### 4.2. Promising Detection Approaches

Extracellular vesicles (EVs), such as exosomes, are now considered possible sources of minimally invasive biomarkers in fields such as oncology, since they include tumour-related nucleic acids, proteins, lipids and other molecular elements. Urinary EVs are especially appealing as urine is easy to obtain and can contain vesicles that are derived from organs like the prostate. In fact, various studies have found molecules associated with prostate cancer (PCa) in urinary EVs, such as PCA3 and TMPRSS2-ERG, which support their use in the non-invasive detection of PCa [55,56,57] tumour. More recent methodological advances have been made in investigating the diagnostic value of extracellular vesicles obtained from other samples, like plasma, expanding the applicability of these exosomes [58].

EV-based approaches have also been explored for HPV detection in PCa. Elghazali et al. compared HPV DNA detection in prostate tissue with plasma- and urine-derived exosomes, supporting the feasibility of using EV-associated viral DNA as a minimally invasive alternative to tissue-based detection [5].

New biosensing technologies could even further enhance both the analytical sensitivity and portability of assays based on extracellular vesicles. Luo et al. created a system based on CRISPR/Cas12a together with a DNA hydrogel for the highly sensitive detection of exosomes, including a portable colorimetric method that allows for visual detection. Even though this method was not designed specifically for HPV or PCa, it shows the potential of CRISPR-based technologies for achieving sensitive and portable detection of extracellular vesicles [59]. More recently, CRISPR/Cas12a-based technology has also been used specifically for PCa-derived exosomes. Miao et al. created a fluorescent and colorimetric dual-modal biosensor that targets PSMA-positive exosomes, showing that PCa-associated exosomes can be sensitively detected and thus supporting the possibility of using CRISPR-based systems for prostate cancer liquid biopsy [60]. Liquid biopsy can also offer additional molecular information in cases where tissue samples are not available or when the DNA obtained from tissue is of poor quality [61].

In other types of cancer, it has been demonstrated that circulating tumour DNA can supply molecular data when the formalin-fixed, paraffin-embedded (FFPE) tissue is degraded or inappropriate for molecular analysis [61]. For instance, Errahhali et al. found in lung adenocarcinoma that liquid biopsy could provide complementary information for EGFR mutation testing, especially in situations where the DNA derived from FFPE tissue was degraded or the amount of tissue available was limited. Nevertheless, this study should be regarded as methodological evidence in support of the complementary value of liquid biopsy and not as direct evidence regarding HPV-associated PCa [62].

Taken as a whole, these findings show that approaches based on extracellular vesicles and liquid biopsies constitute promising methodological advances for the detection of molecular material with high sensitivity in a minimally invasive way. However, their use in the detection of HPV is still in its early stages, and further studies are needed in order to determine whether the HPV nucleic acids associated with extracellular vesicles can serve as reproducible and clinically useful biomarkers.

## 5. HPV and Cancer

### 5.1. HPV, Prostate Cancer Carcinogenesis and Risk Factors

The National Cancer Institute reports that HPV causes around 630,000 of the cancers worldwide, making up about 5% of all cancers. HPV is estimated to cause about 90% of anal cancers, almost all cervical reported cancers, 70% of oropharyngeal cancers, 63% of the penile cancers, 75% of vaginal reported cancers and finally around 69% of vulvar cancers observed [63].

The carcinogenic potential of high-risk HPV is mainly associated with the persistent expression of the viral oncoproteins E6 and E7, which interfere with key cellular mechanisms regulating cell-cycle progression, apoptosis, and cellular senescence. Therefore, E6 and E7 are considered the major HPV oncogenes. E6 promotes the degradation of the tumour suppressor p53 through its interaction with a cellular E3 ubiquitin ligase, resulting in reduced p53-mediated cell-cycle arrest and apoptosis and consequently favouring the survival and proliferation of infected cells. This activity may be complemented by additional interactions with cellular proteins. For example, HPV16 E6 can contribute to increased human telomerase reverse transcriptase (hTERT) expression, supporting cellular immortalization, and E6 has also been reported to interfere with AIF-mediated apoptosis [50,64].

E7 primarily targets the retinoblastoma tumour suppressor protein (pRb), an important regulator of the G1/S transition of the cell cycle. E7 promotes pRb degradation and releases cell-cycle control, thereby favouring progression into S phase and continued proliferation of infected cells [50,64]. E7 can also interact with the related pocket proteins p107 and p130, further contributing to deregulation of cell-cycle control [50,64].

The combined activity of E6 and E7 disrupts complementary tumour-suppressive pathways: E6 weakens cellular responses to DNA damage and apoptotic signals, whereas E7 promotes cell-cycle progression. Persistent expression of these oncoproteins can therefore favour cellular immortalization and transformation, providing a mechanistic basis for HPV-associated carcinogenesis [50,64].

These molecular alterations can contribute to several processes associated with cancer development, including sustained cellular proliferation, evasion of apoptosis, cellular immortalization, and genomic instability. However, the extent to which these mechanisms operate in tissues outside the established HPV-associated cancers remains uncertain [50,64].

The strong association between persistent high-risk HPV infection and several anogenital and oropharyngeal cancers has prompted investigation into whether HPV may also contribute to the development of PCa [65]. However, the established oncogenic mechanisms of E6 and E7 in HPV-associated cancers should not be assumed to occur in prostate tissue. Demonstrating HPV DNA in prostate samples is insufficient to establish that these viral oncogenes are transcriptionally active or that they contribute to prostate carcinogenesis. Evidence such as E6/E7 RNA or protein expression, together with localization of viral material within tumour cells, would provide stronger support for a biologically active infection [50,64].

In 2022, global cancer statistics reported approximately 1.47 million new cases of PCa and 397,000 deaths worldwide. PCa was the second most frequently diagnosed cancer among men and the fifth leading cause of cancer death among men [66].

Several established risk factors for PCa have been described, particularly age, family history and genetic predisposition and ethnicity. Other factors, including lifestyle, metabolic, hormonal, sexual, and inflammatory factors, have also been investigated, although the strength and consistency of the available evidence vary [67]. Key examples are summarized in Table 3.

Despite the identification of several established risk factors, the mechanisms underlying prostate carcinogenesis are not completely understood. In this context, infectious agents have also been investigated as potential contributors to prostate carcinogenesis. Among these, HPV has received particular attention because of its established oncogenic role in several anogenital and oropharyngeal cancers. However, whether HPV infection is associated with PCa development remains controversial [87].

### 5.2. Biological Evidence and Potential Role of HPV in Prostate Carcinogenesis

In 2005, Zoltan Korodi et al., pointed out a possible association between the HPV infection and PCa because of HPV’s previous association with cervical cancer and other anogenital cancers, such as penile and anal cancers [22,88]. However, in their study, they did not observe any significant differences between the detected HPV-16, HPV-18 and HPV-33 in serum and the risk of PCa [22].

Later, in 2011, Y Lin et al., conducted a meta-analysis to investigate the possible correlation between HPV types 16 and 18 and PCa risk and to summarize the results of previous studies. The authors found no significant association between HPV-16 or HPV-18 infection and PCa risk overall. However, subgroup analysis based on HPV-16 DNA detection showed a higher prevalence of HPV-16 in PCa patients (OR = 1.54, 95% CI 1.07–2.20) [26].

Despite these findings, several authors kept searching for a possible correlation between HPV infection and PCa risk partly because of the association with other cancers [22,88] and due to the immortalization of human adult prostatic adenocarcinoma cells by infection with HPV types 16 and 18 [89]. Consequently, numerous case–control studies published over the following years produced conflicting results, with some detecting HPV DNA in prostate cancer tissues [8,9,10,11,12,16,17,18,25], whereas others failed to demonstrate a significant association between HPV infection and PCa [4,13,15,19,20].

In 2023, a meta-analysis compiled case–control studies from four databases, including studies published from 1991 to August 2022. The authors included 27 studies using PCR-based methods to compare HPV detection in PCa samples with either normal prostate tissue or benign prostatic hyperplasia (BPH) as controls. HPV was detected more frequently in PCa than in normal prostate tissue (25.8% vs. 9.2%; OR = 3.07, 95% CI 1.80–5.21) and also more frequently than in BPH tissue (17.4% vs. 10.8%; OR = 1.94, 95% CI 1.43–2.63) [24]. However, the biological meaning of HPV detection depends on the type of evidence obtained. Tissue-based detection of HPV DNA by PCR demonstrates the presence of viral nucleic acid but does not, by itself, establish a transcriptionally active infection or a causal role in prostate carcinogenesis [90]. Similarly, HPV seropositivity reflects previous or current exposure to the virus but does not demonstrate the presence of HPV within prostate tumour cells. Therefore, these findings should be distinguished from evidence of viral activity, such as the detection of E6/E7 transcripts or proteins and the localization of viral sequences within tumour tissue [91].

More recently, evidence has continued to accumulate, although the findings remain heterogeneous. A Japanese study by Shinzawa et al. investigated HPV prevalence and genotype distribution in prostate cancer and seminal vesicle tissues and further assessed viral localization and transcriptional activity. HPV DNA was detected in 9.6% of prostate cancer specimens, with high-risk HPV identified in 4.5% of cases. Importantly, E6/E7 mRNA was detected in 6 of 7 high-risk HPV-positive cases, providing evidence compatible with transcriptionally active HPV infection in a subset of prostate tumours. However, HPV DNA was also detected in non-cancerous prostate lesions, while p16^INK4a^ expression was weak or absent, indicating that these findings do not establish a causal role for HPV in prostate carcinogenesis [6]. Conversely, a study performed in a Pakistani cohort did not detect HPV DNA in prostate cancer specimens, highlighting the variability among populations [4]. More recently, Elghazali et al. reported HPV DNA in prostate tissue as well as in plasma- and urine-derived exosomes. HPV positivity was associated with higher Gleason scores and more advanced T stage, suggesting that exosomal HPV DNA may have potential as a non-invasive biomarker [5]. However, as discussed above, the detection of HPV DNA in exosomes does not demonstrate transcriptionally active infection or a tumour-driving role for HPV.

Although increasing evidence suggests that HPV may play a role in prostate carcinogenesis, the available data remain inconsistent. Several recent studies and meta-analyses have reported positive associations [5,6,23], whereas others have failed to detect HPV DNA or demonstrate an increased risk of prostate cancer [4]. Such discrepancies are likely related to differences in study design, geographical distribution, sample type, HPV detection methods, and the viral genotypes analyzed.

More generally, differences in sample type, tissue sampling, assay sensitivity and specificity, and HPV genotyping methods may contribute to the heterogeneous detection rates reported across studies. The possibility of contamination or detection of transient, incidental or potentially latent viral presence material should also be considered, particularly when HPV DNA is detected without evidence of viral transcription, protein expression, or specific localization within tumour cells [92,93]. Moreover, most available studies are cross-sectional, retrospective, or case–control investigations, limiting the assessment of temporality. Thus, it remains unclear whether HPV infection precedes prostate carcinogenesis, occurs as a consequence of tumour-associated tissue changes, or represents an incidental finding. Therefore, further well-designed multicentre studies using standardized methodologies could provide clarification regarding the biological and clinical relevance of HPV in prostate cancer. Prospective longitudinal studies using standardized protocols are needed to establish whether HPV infection precedes PCa development and to clarify its potential role in prostate carcinogenesis.

Future studies should focus on experimentally testing whether HPV infection can induce persistent oncogenic alterations in prostate cells. Advanced in vitro PCa models incorporating relevant tumour microenvironment components, including immune cells, endothelial cells, cancer-associated fibroblasts, and extracellular matrix, may provide a more physiologically relevant framework for investigating HPV–host interactions and their effects on prostate tumour biology [94]. In particular, co-culture, organoid, and microfluidic models could be used to investigate whether HPV infection is associated with sustained E6/E7 expression, alterations in cell-cycle control and apoptosis, or other persistent phenotypic and molecular changes in prostate cells. Combining these models with the detection of viral DNA, E6/E7 transcripts, and viral proteins could help distinguish transient or incidental HPV detection from biologically active infection and provide stronger evidence regarding a potential causal role of HPV in prostate carcinogenesis.

## 6. Conclusions

HPV is a well-established oncogenic virus in several anogenital and oropharyngeal cancers; however, its potential role in prostate cancer (PCa) remains unresolved. Some studies and meta-analyses report an association between HPV detection and prostate cancer, particularly when HPV DNA is detected in prostate tissue. However, substantial methodological heterogeneity, inconsistent findings, the predominance of observational studies and limited evidence of persistent transcriptionally active infection prevent the conclusion that HPV is a causal factor in prostate carcinogenesis.

Accordingly, HPV infection should not yet be considered an established risk factor for PCa, nor should HPV prevention be considered a strategy to reduce PCa incidence, as there is insufficient evidence to support HPV detection as a routine prognostic or treatment-guiding biomarker. Further well-designed prospective longitudinal studies using standardized protocols are needed to allow comparisons among findings and clarify the causal relevance of HPV in PCa, in order to determine the clinical utility in prostate cancer management.

## Figures and Tables

**Table 1:** Summary of primary studies investigating the association between HPV infection and prostate cancer.

Year	Author	Aim	Subjects	Methodology	Outcomes
**2026**	Ayman et al. [4]	To examine the association between HPV infection and renal and prostate cancers in the Pakistani population.	50 renal cancer and 50 prostate cancer biopsy samples.	HPV DNA detection by conventional PCR (GP5+/6+), followed by HPV16/18 genotyping and Sanger sequencing.	HPV was detected in 30% (15/50) of renal cancer samples, with 93% showing HPV16/18 co-infection. All prostate cancer samples were HPV-negative, showing no association between HPV and prostate cancer.
**2026**	Elghazali et al. [5]	To investigate HPV DNA detection in prostate tissue and plasma- and urine-derived exosomes and its association with clinicopathological features of PCa.	50 PCa patients and 50 age-matched BPH controls.	HPV DNA detection by nested PCR in prostate tissue and plasma- and urine-derived exosomes.	HPV DNA was detected in 32% of prostate tissues, 24% of plasma-derived exosomes, and 28% of urine-derived exosomes, compared with 2% of controls. HPV positivity was associated with higher Gleason score and advanced T stage.
**2025**	Shinzawa R. et al. [6]	To determine HPV prevalence and genotype distribution in prostate cancer and seminal vesicle tissues and investigate its potential role in prostate carcinogenesis.	157 FFPE prostate cancer specimens from 156 primary lesions and 1 metastatic lesion, plus 156 seminal vesicle specimens.	Nested PCR and HPV genotyping; HPV-DNA localization by ISH; E6/E7 mRNA ISH and p16^INK4a^ IHC in HR-HPV-positive cases.	HPV-DNA was detected in 9.6% of PCa tissues and 0% of seminal vesicles; HR-HPV was detected in 4.5%. HPV prevalence was higher in high-grade tumours. E6/E7 mRNA was expressed in 6/7 HR-HPV-positive cases, whereas p16^INK4a^ expression was weak or absent.
**2023**	M Mikhaleva et al. [7]	To assess the relationship between viral infection and prostate cancer and its clinical and morphological features.	50 PCa and 48 BPH patients.	Real-time PCR of surgical prostate specimens for HPV, HSV-1/2, CMV, EBV and HHV-6.	HPV was investigated in prostate tissues, but no specific HPV prevalence or significant association with PCa was reported. Overall viral detection was higher in PCa than BPH (50.0% vs. 31.3%).
**2023**	Tobias Nellessen et al. [8]	To characterize HPV infection in patients with PCa undergoing radical prostatectomy.	140 PCa patients undergoing radical prostatectomy.	HPV DNA detection by PCR; positive samples were genotyped by LCD-Array and assessed by p16 IHC. HPV-16 L1 antibodies were also measured.	HPV DNA was detected in 9.3% (13/140) of prostatectomy specimens, with HPV16 as the most frequent subtype (5/13). No significant association was found between HPV status and clinical or tumour characteristics.
**2023**	Soukayna Alaoui Sosse et al. [9]	To investigate the presence of HPV DNA in the blood of patients with PCa and its association with clinicopathological characteristics.	100 PCa patients and 50 controls.	HPV DNA detection in blood by nested PCR targeting the L1 gene.	HPV DNA was detected in 10% of PCa patients and in none of the controls. HPV-positive cases were limited to stages I–II, with no significant association with Gleason score or other evaluated clinical characteristics.
**2023**	M. Yahya Ahmed et al. [10]	To assess the presence and expression of 12 high-risk HPV types in benign and malignant prostate tissues.	49 fresh prostate specimens: 14 adenocarcinoma and 35 benign samples.	PCR and Sanger sequencing for 12 HR-HPV types; HPV E7 expression assessed by IHC.	HR-HPV DNA was detected in 35.7% (5/14) of adenocarcinoma and 31.4% (11/35) of benign samples, with no significant association between HR-HPV status and pathological status (*p* = 0.063). HPV35 was the most frequent genotype. HPV E7 was expressed in all HPV-DNA-positive samples.
**2023**	Heng-Jui Chang et al. [11]	To evaluate potential risk factors for PCa, including HPV infection, family history, dietary factors and sexual behaviour.	143 PCa patients and 135 BPH controls, aged 55–86 years.	Matched case–control study using a structured questionnaire and HPV DNA detection in paraffin-embedded/formalin-fixed prostate tissues by commercial HPV testing and genotyping kits.	HPV DNA was detected in 7.0% of PCa patients and 1.5% of BPH controls, and HPV infection was identified as a potential risk factor for PCa.
**2023**	Nalisson Marques Pereira et al. [12]	To evaluate the presence of HPV in neoplastic and non-neoplastic prostate tissue and its possible association with PCa.	162 patients undergoing prostate biopsy; after combining biopsy and immunohistochemistry results, 95 had PCa, 55 had no cancer, and 7 had an inconclusive diagnosis.	Prostate tissue obtained by transrectal ultrasound-guided biopsy. HPV DNA was detected by real-time PCR targeting the L1 gene.	HPV was detected in 7/95 (7.4%) PCa patients and 2/55 (3.6%) patients without cancer, with none detected in inconclusive cases. No significant association was found between HPV presence and PCa (*p* = 0.487).
**2017**	Xiaojun Zhao et al. [13]	To explore the serological association between HPV16 infection and PCa using a peptide microarray.	75 untreated PCa patients and 80 controls.	Serum HPV16 antibodies were assessed using a peptide microarray containing 241 HPV16-derived peptides.	HPV16 seropositivity was detected in 64.0% of PCa patients vs. 17.5% of controls (*p* < 0.01). The assay showed an AUC of 0.793 (95% CI 0.721–0.864), and HPV16 antibody positivity was significantly associated with higher Gleason scores.
**2013**	Lin Huang et al. [14].	To assess the association between high-risk HPV16/18 infection and PCa and its relationship with clinicopathological characteristics.	75 PCa patients and 73 BPH controls.	HPV16/18 detection in prostate tissue by immunohistochemistry and PCR combined with reverse dot blot (RDB) assay.	HPV16/18 positivity was higher in PCa than BPH by both immunohistochemistry (21.3% vs. 9.5%, *p* = 0.049) and PCR/RDB. By PCR/RDB, HPV16 was detected in 22.6% vs. 8.2% and HPV18 in 17.8% vs. 4.1% of PCa and BPH samples, respectively (*p* = 0.001). HPV16/18 infection was associated with clinical T stage and Gleason score.
**2016**	Sonja Lumme et al. [15]	To evaluate the individual and combined effects of HPV-18, *Chlamydia trachomatis*, enterolactone, vitamin D and free testosterone on the risk of PCa.	483 PCa cases and 1055 controls with complete data from a joint cohort of 209,000 healthy men.	Population-based longitudinal study using serum samples from three Nordic biobanks. HPV-18 was assessed using serological biomarkers, and associations with PCa risk were evaluated using multivariate regression analyses.	No significant association was found between HPV-18 exposure and PCa risk.
**2015**	Neha Singh et al. [16]	To determine the prevalence of high-risk HPV infection in PCa and its association with clinicopathological characteristics in an Indian population.	95 histopathologically confirmed PCa patients and 55 BPH controls.	HPV DNA detection by consensus-primer PCR, followed by type-specific PCR for high- and low-risk HPV types.	HPV infection was detected in 41% of PCa samples and 20% of BPH controls. HPV16 was detected in 32% of PCa vs. 5% of BPH samples (*p* = 0.0004), while HPV18 was detected in 6% of PCa samples. HPV infection was associated with advanced tumour stage (III–IV) and Gleason score ≥ 8.
**2013**	Mariarosa Pascale et al. [17]	To examine the prognostic impact of HPV infection on survival in patients with primary PCa.	150 patients with primary PCa, diagnosed between 1992 and 1994.	HPV E7 protein expression was assessed in formalin-fixed, paraffin-embedded tumour tissue by immunohistochemistry. HPV DNA was analyzed by PCR and sequencing in a subset of samples.	HPV E7 expression was detected in 74.67% (112/150) of patients. HPV-positive patients had significantly shorter overall survival than HPV-negative patients (4.59 vs. 8.24 years, *p* = 0.0381). HPV status remained an independent predictor of overall survival in multivariate analysis (HR = 1.57, 95% CI 1.03–2.39, *p* = 0.034). HPV16 was identified in the sequenced samples.
**2012**	Noel J Whitaker et al. [18]	To investigate the presence of high-risk HPV and EBV in normal, benign and malignant prostate tissues and their potential role in PCa.	150 prostate tissue specimens: 50 normal, 50 benign and 50 malignant tissues.	HPV and EBV detection by in situ PCR and conventional PCR followed by sequencing. Histological assessment was also performed to identify koilocytes.	HPV18 sequences were detected in 58% of PCa tissues, 16% of benign tissues and 16% of normal tissues. Koilocytes were identified in 24% of PCa specimens, and HPV18 sequences were localized to nuclei. The findings suggested a potential oncogenic role for HPV18 in PCa.
**2010**	Siobhan Sutcliffe et al. [19]	To evaluate the association between HPV16, HPV18 and HPV31 serostatus and PCa risk.	616 PCa cases and 616 controls from the Prostate Cancer Prevention Trial (PCPT).	Nested case–control study. Serum IgG antibodies against HPV16, HPV18 and HPV31 were assessed by type-specific ELISA.	No significant association was observed between HPV16, HPV18 or HPV31 seropositivity and PCa risk. For strong seropositivity, ORs were 1.07 (95% CI 0.77–1.48) for HPV16, 0.87 (95% CI 0.47–1.63) for HPV18 and 1.15 (95% CI 0.80–1.64) for HPV31.
**2008**	Wen-Yi Huang et al. [20]	To investigate the association between sexually transmissible infections, including HPV16 and HPV18, and PCa risk.	868 PCa cases and 1283 controls, matched by race, age, time since initial screening and year of blood draw.	Nested case–control study within the PLCO Cancer Screening Trial. Serum antibodies against HPV16 and HPV18 and other sexually transmissible infections were assessed using serological assays.	No significant association was found between HPV16 or HPV18 antibodies and PCa risk, although men with one or more STIs showed a borderline increased risk of PCa (OR = 1.3, 95% CI 1.0–1.6).
**2007**	Siobhan Sutcliffe et al. [21]	To assess the association between antibodies against HPV16, HPV18 and HPV33 and the risk of PCa.	691 PCa cases and 691 matched controls from the Health Professionals Follow-up Study.	Nested case–control study. Plasma antibodies against HPV16, HPV18 and HPV33 were assessed using enzyme-based immunoassays.	No significant association was found between HPV16, HPV18 or HPV33 seropositivity and PCa risk. The ORs were 0.83 (95% CI 0.57–1.23) for HPV16, 1.04 (95% CI 0.66–1.64) for HPV18 and 1.14 (95% CI 0.76–1.72) for HPV33.
**2005**	Zoltan Korodi et al. [22]	To investigate the association between HPV16, HPV18 and HPV33 infection and the risk of PCa.	804 PCa cases and 4 matched controls per case from three Nordic biobanking cohorts.	Nested case–control study. Serum IgG antibodies against HPV16, HPV18 and HPV33 were assessed by standard ELISA.	The combined seroprevalence of HPV16/18/33 was 13.4% in PCa cases and 14.0% in controls, with no significant association with PCa risk (OR = 0.94, 95% CI 0.74–1.19). No noteworthy differences were observed according to HPV type, country or antibody level.

**Table 2:** Summary of systematic reviews and meta-analyses investigating HPV and prostate cancer.

Year	Author	Aim	Subjects	Methodology	Outcomes
**2025**	Arriaga-Izabal et al. [23]	To systematically evaluate the association between HPV infection in prostate tissue and PCa in Mexican men and to assess the prevalence of high- and low-risk HPV genotypes.	8 case–control studies, comprising 1059 PCa cases and 1768 controls from the Mexican population.	Systematic review and meta-analysis of studies identified through PubMed and Web of Science. Study quality was assessed using the Newcastle–Ottawa Scale, and pooled estimates were calculated using a random-effects model.	HPV infection in prostate tissue was significantly associated with PCa (OR = 2.34, 95% CI 1.52–3.60). High-risk HPV accounted for 77% (95% CI 69–84%) of HPV-positive prostate samples, compared with 23% (95% CI 16–31%) for low-risk HPV. No significant publication bias or heterogeneity was detected.
**2023**	Irina A. Tsydenova et al. [24]	To systematically evaluate the association between HPV infection and PCa.	27 case–control studies, comprising 1607 PCa tissue samples and 1515 control samples (317 normal prostate tissues and 1198 BPH tissues).	Systematic review and meta-analysis of case–control studies published from 1991 to 2022. HPV was detected by PCR, and pooled estimates were calculated using meta-analytic methods; heterogeneity and publication bias were assessed using Cochran’s Q test, I^2^, Begg’s and Egger’s tests.	HPV was detected more frequently in PCa than in normal prostate tissue (25.8% vs. 9.2%; OR = 3.07, 95% CI 1.80–5.21). In comparison with BPH, HPV was detected in 17.4% of PCa vs. 10.8% of BPH samples, with a pooled OR = 1.94 (95% CI 1.43–2.63).
**2014**	Somayeh Jalilvand et al. [25]	To assess the prevalence of HPV infection and HPV types in different cancers and HPV-related diseases in the Iranian population.	61 studies covering different cancer types and HPV-related diseases in Iranian populations.	Systematic assessment of published studies. HPV prevalence and genotype distribution were extracted for different cancer types and control groups.	HPV was detected in 10.4% of prostate cancer cases (24/231) and 3.8% of BPH controls (17/446). HPV16 and HPV18 were the most frequently detected types overall; in prostate cancer, their detection was approximately similar between cases and controls.
**2011**	Y. Lin et al. [26]	To evaluate the association between HPV16 or HPV18 infection and PCa risk.	25 eligible studies investigating HPV16; 13 of these studies also provided data on HPV18.	Meta-analysis of studies evaluating HPV16/18 infection in relation to PCa. Pooled ORs with 95% CIs were calculated using fixed- or random-effects models, with stratified analyses according to HPV detection method and geographic region.	No significant association was found between HPV16 and PCa risk (OR = 1.09, 95% CI 0.97–1.23) or between HPV18 and PCa risk (OR = 1.05, 95% CI 0.89–1.24). However, in the subgroup analysis based on HPV16 DNA detection, HPV16 prevalence was higher in PCa (OR = 1.54, 95% CI 1.07–2.20).

**Table 3:** Established risk factors and possible or controversial associations with prostate cancer.

Risk	Evidence	Findings
**Age**	Established	PCa risk increases substantially with age, particularly after 50 years [68].
**Family history and genetic predisposition**	Established	Family history and inherited genetic variants, including DNA-repair gene alterations, are associated with increased PCa risk [69,70,71,72].
**Ethnicity**	Established	PCa incidence varies across populations, with particularly high incidence reported among Black/African American men [73,74,75].
**Smoking and alcohol**	Possible / heterogeneous	Smoking is consistently associated with PCa mortality and recurrence [76]. Alcohol consumption has been demonstrated to increase PCa incidence, mortality and prevalence [77].
**Obesity and metabolic syndrome**	Possible / heterogeneous	Associations have been reported with PCa incidence, mortality, and disease outcomes, but the strength of these associations varies [78,79].
**Physical activity**	Unclear	The relationship between physical activity and PCa risk remains uncertain, although higher activity levels have been associated with lower risk in some studies [80].
**Diet and nutrition**	Controversial	Associations between specific dietary factors or supplements and PCa risk remain under investigation [81,82].
**Medications**	No consistent association	Available evidence does not support a significant association between antidiabetic medication use and PCa risk [83].
**Sex and vasectomy**	Possible association	Vasectomy has been associated with long-term PCa risk, while higher ejaculation frequency has been associated with lower PCa risk [84].
**Hormones**	Possible association	Associations between circulating hormone levels and PCa risk have been reported, including lower risk with low free testosterone and higher risk with elevated IGF-I levels [85].
**Infection, inflammation, and chemokines**	Possible	Inflammatory pathways and chemokines may contribute to prostate tumour biology, including proliferation, angiogenesis, and metastasis [86].

## Data Availability

No new data were created or analyzed in this study.

## Abbreviations

The following abbreviations are used in this manuscript:

AIF	Apoptosis-inducing factor
ATP	Adenosine triphosphate
BPH	Benign prostatic hyperplasia
CI	Confidence interval
CMV	Cytomegalovirus
DISC	Death-inducing signalling complex
DNA	Deoxyribonucleic acid
EBV	Epstein–Barr virus
FFPE	Formalin-Fixed, Paraffin-Embedded
EGFR	Epidermal growth factor receptor
ELISA	Enzyme-linked immunosorbent assay
HPV	Human papillomavirus
LCR	Long control region
MHC1	Major histocompatibility complex class I
ORF	Open reading frame
PCa	Prostate cancer
PCR	Polymerase chain reaction
STDs	Sexually transmitted diseases
TR-FIA	Time-resolved fluoroimmunoassay
TRAIL	TNF-related apoptosis-inducing ligand
URR	Upstream regulatory region
VEGF	Vascular endothelial growth factor
VLPs	Virus-like particles
